# Effect of exercise training on skeletal muscle protein expression in relation to insulin sensitivity: Per‐protocol analysis of a randomized controlled trial (GO‐ACTIWE)

**DOI:** 10.14814/phy2.14850

**Published:** 2021-05-27

**Authors:** Lea Bruhn, Rasmus Kjøbsted, Jonas Salling Quist, Anne Sofie Gram, Mads Rosenkilde, Kristine Færch, Jørgen F.P. Wojtaszewski, Bente Stallknecht, Martin Bæk Blond

**Affiliations:** ^1^ Department of Biomedical Sciences Faculty of Health and Medical Sciences University of Copenhagen Copenhagen Denmark; ^2^ Steno Diabetes Center Copenhagen Gentofte Denmark; ^3^ Section of Molecular Physiology August Krogh Club Department of Nutrition, Exercise and Sports University of Copenhagen Copenhagen Denmark

**Keywords:** exercise intensity, exercise training, insulin sensitivity, PDH

## Abstract

Exercise training improves peripheral insulin sensitivity and leads to molecular adaptations in the skeletal muscle. We investigated changes in the expression of key muscle proteins in the glucose metabolic pathway following active commuting by bike or leisure‐time exercise at two different intensities. In addition, potential associations between insulin sensitivity and muscle protein expression were examined. This per‐protocol analysis included 72 out of 130 physically inactive, healthy women and men (20–45 years) with overweight/obesity (BMI: 25–35 kg/m^2^) who completed 6 months of no intervention (CON, *n *= 12), active commuting by bike (BIKE, *n *= 14), or leisure‐time exercise of moderate (MOD, *n *= 28) or vigorous (VIG, *n *= 18) intensity. Exercise was prescribed 5 days/week with a weekly exercise energy expenditure of 1,600 kcal for women and 2,100 kcal for men. Insulin sensitivity was determined by a hyperinsulinemic euglycemic clamp and skeletal muscle biopsies were obtained from m. vastus lateralis and analyzed for protein expression at baseline and after 3 and 6 months of intervention. We found an increased expression of pyruvate dehydrogenase (PDH) in the exercise groups compared with the control group following 6 months of training. No differential effects were observed on the protein expression following moderate versus vigorous intensity exercise. In addition, we found a positive association between insulin sensitivity and the expression of glucose transporter type 4 as well as PDH. The positive association and the increase in expression of PDH after exercise training points toward a role for PDH in the training‐induced enhancement of insulin sensitivity.

## Graphical abstract

Associations between the expression of key muscle glucoregulatory proteins and insulin sensitivity, indicate that pyruvate dehydrogenase regulates insulin sensitivity after exercise training in humans.

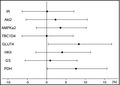

## 1 INTRODUCTION

Exercise improves insulin sensitivity and may contribute to the prevention of type 2 diabetes (Pedersen & Saltin, 2015). However, the molecular mechanisms underlying the exercise training‐induced increase in peripheral insulin sensitivity are complex and not fully elucidated.

Insulin‐stimulated glucose uptake is initiated by insulin binding to and activating the insulin receptor (IR) on the muscle plasma membrane (Wojtaszewski et al., 1999). This initiates a signaling cascade leading to translocation of type 4 glucose transporter (GLUT4) to the muscle plasma membrane, thereby increasing the potential for glucose uptake into skeletal muscle (Leto & Saltiel, 2012). Knockout of IR in mouse skeletal muscle has been shown to inhibit insulin‐stimulated glucose uptake (Wojtaszewski et al., 1999) while IR overexpression improves whole‐body glucose tolerance (Wang et al., 2020). In the proximal insulin signaling cascade the Rac‐beta serine/threonine‐protein kinase 2 (Akt2) is vital for insulin‐stimulated glucose uptake in the skeletal muscle (Cho, 2001), and overexpression of constitutively active Akt2 in rat muscle has been shown to increase glucose uptake (Cleasby et al., 2007). The Akt2 downstream target TBC1 domain family member 4 (TBC1D4) regulates GLUT4 translocation to the muscle plasma membrane in response to insulin (Kramer et al. 2006). Although it functions as an inhibitor of GLUT4 translocation, evidence suggests that the expression of TBC1D4 is important for maintaining muscle insulin sensitivity as it prevents lysosomal degradation of GLUT4 (Xie et al. 2016). TBC1D4 is also targeted by the exercise‐activated AMP‐activated protein kinase alpha 2 subunit (AMPKα_2_) and we have previously shown that this interaction enhances muscle insulin sensitivity (Kjøbsted et al., 2015, 2017, 2019). The ability of insulin to increase glucose uptake into skeletal muscle is highly dependent on GLUT4 (Zisman et al., 2000), and in mice overexpression of GLUT4 in the skeletal muscle has been shown to increase insulin‐stimulated glucose uptake (Ren et al., 1995). Once glucose has entered the muscle cell, it is metabolized to glucose‐6‐phosphate by hexokinase II (HKII) whereby the glucose gradient across the muscle plasma membrane is maintained. Overexpression and partial knockout of HKII in the skeletal muscle increases and decreases insulin‐stimulated glucose uptake, respectively, indicating that the expression of HKII is important for the regulation of insulin‐stimulated glucose uptake in skeletal muscle (Fueger et al., 2004, 2007). Following phosphorylation by HKII, glucose is mainly directed towards storage and oxidation in skeletal muscle. Glycogen synthase (GS) is the rate‐limiting enzyme for the storage of glucose as glycogen, and dysregulation of GS has been associated with insulin resistance in skeletal muscle (Hojlund et al., 2003; Skurat et al., 1996). Pyruvate dehydrogenase (PDH) is responsible for the first step in the conversion of pyruvate to acetyl‐CoA in mitochondria, thereby acting as a central regulator of glucose oxidation (Abdul‐Ghani et al., 2011). It has been proposed that impaired PDH function plays a causative role in the development of skeletal muscle insulin resistance and that exercise can remedy this impairment (Constantin‐Teodosiu, 2013). Smaller non‐randomized studies have reported increased expression of some of these key muscle proteins in the glucose metabolic pathways following exercise training coinciding with increased insulin sensitivity, but the findings have been inconsistent (Biensø et al., 2015; Dela et al., 1993; Frøsig et al., 2007; Steenberg et al., 2019; Vind et al., 2011). This inconsistency is likely both attributable to differences in study design and a product of the increased risk of false negative findings and the higher false positive rates that characterize small studies.

Collectively, evidence suggests that the expression of the above‐mentioned proteins could be important for insulin‐stimulated glucose uptake in skeletal muscle. However, it remains uncertain whether the expression of these proteins in the glucose metabolic pathways is all responsive to exercise training and whether their expression associates with insulin sensitivity in humans. In addition, the optimal training prescription to improve insulin sensitivity is still debated (Bird & Hawley, 2017; McGarrah et al., 2016; Reichkendler et al., 2014). It has been proposed that the adaptations leading to increased insulin sensitivity following exercise training depends on the exercise intensity (McGarrah et al., 2016); however, it has not been investigated whether this includes differential changes in the expression of the proteins assessed in this paper.

We previously reported that 6 months of active commuting by bike or leisure‐time exercise, of either moderate or vigorous intensity, produced similar improvements in insulin sensitivity measured by a hyperinsulinemic euglycemic clamp in previously physically inactive women and men with overweight or obesity (Blond et al., 2019). To examine the association between muscle protein expression and insulin sensitivity and add further to the discussion regarding the impact of exercise intensity on the molecular mechanisms responsible for the training‐induced changes in insulin sensitivity, we hereby report the findings for the following eight key proteins within the glucose metabolic pathways in skeletal muscle: IR, Akt2, TBC1D4, AMPKα_2_, GLUT4, HKII, GS, and PDH. Based on the evidence presented above, we hypothesized that the expression of one or more of these proteins in skeletal muscle are associated with insulin sensitivity and that the molecular mechanisms mediating the exercise training‐induced changes in insulin sensitivity are independent of exercise intensity.

## 2 MATERIALS AND METHODS

### 2.1 Participants and study design

We performed an exploratory analysis based on per protocol completers from the GO‐ACTIWE (Governing Obesity: Active Commuting To Improve health and Wellbeing in Everyday life, http://go.ku.dk) trial. GO‐ACTIWE was a 6‐month single‐center multi‐arm parallel‐group randomized controlled exercise trial performed at the Department of Biomedical Sciences, University of Copenhagen. The study was approved by the ethics committee of the Capital Region of Denmark (H‐4‐2013‐108), registered at clinicaltrials.gov (ID‐code: NCT01962259) and adhered to the Helsinki Declaration. Recruitment of participants and data collection took place from October 2013 until June 2016. Written and verbal informed consent was obtained from all participants prior to inclusion in the study.

The trial was designed to investigate the health benefits of active commuting by bike or leisure‐time exercise of either moderate or vigorous intensity; and dimensioned to detect changes in insulin sensitivity (primary outcome), visceral fat mass, total fat mass, cardiorespiratory fitness, energy balance, endogen thrombin potential, and tissue‐type plasminogen activator (Rosenkilde et al., 2017). The results for these outcomes and others have been published (Blond et al., 2019; Gram et al., 2017, 2018; Kern et al., 2020; Quist, Blond, et al., 2019; Quist, Rosenkilde, et al., 2019; Quist et al., 2018). The trial, including randomization procedure, has been described in detail elsewhere (Blond et al., 2019; Rosenkilde et al., 2017). In brief, 130 younger (20–45 years), physically inactive (regular exercise <2 h/week and active commuting <25 km/week; VO_2_peak: women <40 ml O_2_/min/kg and men <45 ml O_2_/min/kg), non‐smoking, Caucasian women and men with overweight or class 1 obesity (BMI 25–35 kg/m^2^; fat percentage: women ≥32% and men ≥25%) were recruited. Exclusion criteria were: blood pressure >140/90 mmHg, fasting blood glucose >6.1 mmol/l, first‐degree relatives with type 2 diabetes and regular use of medication (excluding oral contraceptives). Participants were randomized in a 1:2:2:2 ratio to one of the following four groups: control (CON, maintenance of habitual lifestyle, *n *= 18), active commuting by bike (BIKE, self‐selected intensity, *n *= 35), or leisure‐time exercise at moderate (MOD, 50% VO_2_peak reserve, *n *= 39) or vigorous (VIG, 70% VO_2_peak‐reserve, *n *= 38) intensity. Out of these 72 participants completed per protocol and had muscle biopsy material available at baseline and from at least one of the follow‐up time points: CON: *n* = 12; BIKE: *n* = 14; MOD: *n* = 28 and VIG: *n* = 18.

### 2.2 Interventions

For all exercise groups, exercise was prescribed 5 days/week with a total weekly exercise energy expenditure of 1,600 kcal in women and 2,100 kcal in men. Participants in BIKE were instructed to commute by bike to and from work/school whereas participants in MOD and VIG were instructed to perform aerobic exercise (e.g., walking, running, rowing, cross trainer, or stationary cycling) at a heart rate corresponding to 50% or 70% of VO_2_peak‐reserve, respectively. All exercise sessions were monitored with the use of heart rate monitors (Polar RC3 GPS, Polar, Finland) and individually adjusted after 6 weeks and 3 months based on changes in maximal heart rate, VO_2_peak and body weight. Exercise energy expenditure for each exercise session was calculated by multiplying the estimated average oxygen uptake during the exercise session with the energy equivalent of oxygen and the exercise duration. The estimated average oxygen was determined by constructing a first‐degree equation between oxygen uptake and heart rate during three submaximal workloads performed in the warm‐up period of the fitness test closest in time to the exercise session; secondly the average heart rate of the individual exercise session was used to estimate the average oxygen uptake of the given session. Which value of the energy equivalent of oxygen to be used for the given exercise session was determined based on the respiratory exchange rate recorded when the heart rate during the fitness test was equivalent to the average heart rate of the exercise session. Participants were instructed to upload training data (www.polarpersonaltrainer.com) every week to facilitate and monitor exercise adherence. Per protocol completers were defined as those who adhered to 80%–120% of the prescribed exercise energy expenditure measured by the heart rate monitor.

### 2.3 Measurements

#### 2.3.1 Body weight

Body weight was measured (SECA 767; Vogel&Halke) after an overnight fast (≥10 h) with the participants wearing light clothes. The measurement was performed at baseline and after 3 and 6 months of intervention.

#### 2.3.2 Cardiorespiratory fitness

Cardiorespiratory fitness was determined as VO_2_peak during an incremental test performed on an electronically braked cycle (Lode Excalibur) and using open circuit indirect respiratory calorimetry (Oxycon Pro; Jaeger). The test included a 9‐min warm‐up period after which the workload was increased every min by 20 Watt for women and 25 Watt for men until exhaustion. Attainment of VO_2_peak was accepted when a levelling‐off in oxygen consumption was observed despite increasing workload or as subjective exhaustion combined with a respiratory exchange ratio >1.15. Cardiorespiratory fitness tests were performed at baseline and after 6 weeks and 3 and 6 months of intervention.

#### 2.3.3 Insulin sensitivity

Insulin sensitivity was determined by a 120‐min hyperinsulinemic euglycemic clamp using previously described procedures (Reichkendler et al., 2014). In brief, hyperinsulinemia was obtained by a primed continuous infusion of 40 mU/m^2^/min exogenous human insulin (Actrapid, 100 IU/ml; Novo Nordisk). Plasma glucose was measured every 5 min and euglycemia was maintained by a variable glucose infusion. Arterialized blood samples were obtained at 30, 60, 90, and 120 min for measurements of plasma glucose and insulin concentrations. Insulin sensitivity was defined as the glucose infusion rate from 90 to 120 min corrected for variations in the glucose concentration in the extracellular distribution space (Muniyappa et al., 2008) and divided by the steady state plasma insulin concentration. The insulin clamp was performed at baseline and after 3 and 6 months of intervention. Participants in the exercise groups were instructed to perform the last exercise bout 36–48 h before testing.

#### 2.3.4 Muscle processing and western blotting

Muscle biopsies from m. *vastus lateralis* were obtained under local anesthesia (3–5 ml of xlocaine, 20 mg/ml) in the fasted and non‐insulin‐stimulated state using the Bergström needle biopsy technique (Bergström, 1975). Muscle specimens were snap‐frozen in liquid nitrogen and stored at −80°C for further analysis. Muscle biopsies were taken at baseline and after 3 and 6 months of intervention.

Muscle biopsies were freeze‐dried and dissected free of visible fat, blood, and connective tissue before being homogenized in ice‐cold buffer (10% glycerol, 20 mmol/L sodium pyrophosphate, 1% NP‐40, 2 mmol/L phenylmethylsulfonyl fluoride, 150 mmol/L sodium chloride, 50 mmol/L HEPES, 20 mmol/L b‐glycerophosphate, 10 mmol/L sodium fluoride, 1 mmol/L EDTA, 1 mmol/L EGTA, 10 mg/mL aprotinin, 3 mmol/L benzamidine, 10 mg/ml leupeptin, and 2 mmol/L sodium ortovanadate, pH 7.5) for 2 × 30 s at 30 Hz using steel beads and TissueLyzer II (Qiagen). Homogenates were rotated end over end for 1 hour before centrifuged at 16,000 *g* for 20 min at 4°C. The lysate was collected, and the total protein abundance was determined by the bicinchoninic acid method (ThermoFisher Scientific). Muscle lysate was prepared in Laemmli buffer and heated for 10 min at 96°C. Equal amounts of protein were loaded on 8% or 10% self‐cast gels, separated by SDS‐PAGE and transferred to polyvinylidene fluoride membranes using semidry blotting. Membranes were blocked for 10 min in 2% skim milk and probed overnight at 4°C with a primary antibody. On the following day, membranes were incubated with a secondary antibody for 45 min at room temperature and proteins with bound antibody were visualized with chemiluminescence (Milipore) using a digital imaging system (ChemiDoc, MP System; Bio‐Rad).

#### 2.3.5 Antibodies

Antibodies against Akt2 (catalog #3063) and HKII (catalog #2867) were purchased from Cell Signaling Technology. Anti‐ GLUT4 (catalog #PA1‐1065; Thermo Fisher Scientific); Anti‐IR (kindly provided by Professor Ken Siddle, Cambridge University, Cambridge, UK); anti‐TBC1D4 (catalog #189890, Abcam); anti‐AMPKα_2_ (catalog #SC‐19131, Santa Cruz Biotechnology); Anti‐GS (kindly provided by Professor Oluf Pedersen, University of Copenhagen, Copenhagen, Denmark); anti‐PDH (custom made, Professor D.G. Hardie, University of Dundee, Dundee, Scotland, UK).

### 2.4 Statistical analysis

All missing data were assumed to be missing at random. Descriptive data are presented as the median and interquartile range. Comparison of exercise compliance between the three exercise groups was performed using ANOVA with post hoc *t*‐test to compare the individual groups in case of a significant *F*‐test.

Analyses of intervention effects were performed using a linear mixed model with the outcomes as a function of group, time, and time × group. The differences in delta values between the intervention groups and the control group were estimated using the linear mixed model (mean estimate at follow‐up_intervention group_−mean estimate at baseline_intervention group_)−(mean estimate at follow‐up_control group_−mean estimate at baseline_control group_). Additional differences in delta between MOD and VIG were calculated. Sex was included in the model for the analysis of insulin sensitivity and cardiorespiratory fitness as sex differences were expected for these outcomes. The analysis of the associations between insulin sensitivity and protein expression (across all timepoints) was performed using a linear mixed model in which insulin sensitivity was predicted from time, protein expression level, time x protein expression level, and sex. Protein expression levels were standardized to a mean of 0 and a standard deviation of 1 prior to the association analyses. This allows a direct comparison of the strength of the association across predictors (Aarts et al., 2014), in this case the direct comparison of the strength of the association between insulin sensitivity and the expression of individual proteins. For all linear mixed models, a repeated statement was defined for time on participant level and an unstructured covariance structure was applied.

Model fit was evaluated by visual inspection of the distribution of residuals. Data representing insulin sensitivity, Akt2, AMPKα_2,_ and HKII were logarithmically transformed (natural logarithm) to fit the distributional assumptions of the statistical analysis. The results for these outcomes were back‐transformed for the presentation of intervention effects and presented as the relative difference between the relative changes from baseline to follow‐up within the two groups being compared, whereas results for the association analyses are presented on log‐scale. The results from the mixed linear models were expressed as estimated mean differences with 95% confidence intervals (95% CI) for group comparisons and means averaged over covariates with 95% CI for levels at a given time point. Statistical significance was defined as *p *< 0.05. All statistical analyses were performed in SAS version 9.4 (SAS Institute).

## 3 RESULTS

### 3.1 Participant characteristics

Out of the 72 participants included in this per protocol analysis three participants in MOD and one in VIG were missing protein expression data at the 3 months follow‐up visit. At the 6 months follow‐up visit, two participants in CON, BIKE, and MOD, plus one in VIG, were missing protein expression data. Baseline participant characteristics are presented in Table 1.

**TABLE 1:** Baseline characteristics of study participants

	CON (*n* = 12)	BIKE (*n* = 14)	MOD (*n* = 28)	VIG (*n* = 18)
Sex, F/M (*N*)	6/6	8/6	11/17	7/11
Age (years)	37 (28; 42)	35 (27; 42)	33 (28; 38)	37 (30; 41)
Body weight (kg)	93.0 (90.3; 101.5)	90.0 (81.5; 100.4)	92.9 (79.7; 97.2)	90.7 (81.7; 104.7)
BMI (kg/m^2^)	30 (28; 32)	29 (28; 31)	29 (28; 30)	30 (28; 32)
Fat free mass (kg)	60.9 (48.6; 68.8)	55.9 (46.6; 61.5)	59.9 (44.7; 65.9)	62.1 (44.4; 69.6)
Fat (%)	34.4 (33.1; 43.0)	41.2 (28.8; 45.9)	34.7 (30.8; 42.3)	36.8 (30.8; 44.0)
VO_2_peak (ml/min)	3068 (2117; 3543)	2759 (2252; 3158)	2828 (2297; 3183)	2810 (2440; 3130)
Cardiorespiratory fitness (ml/min/kg)	30.5 (23.8; 35.6)	30.8 (27.3; 34.1)	30.8 (27.0; 33.4)	30.6 (26.2; 32.4)
Fasting plasma glucose (mmol/L)	5.3 (4.9; 5.6)	5.2 (5.0; 5.6)	5.2 (5.0; 5.5)	5.4 (5.1; 5.7)
Insulin sensitivity (mg/min/pM)	0.67 (0.49; 0.82)	0.66 (0.47; 0.98)	0.66 (0.52; 0.75)*	0.63 (0.47; 0.96)

Data are presented as median (25th and 75th percentile) or number (*N*). Insulin sensitivity: glucose infusion rate measured by the hyperinsulinaemic euglycaemic clamp divided by insulin levels during the clamp. *n = 27.

Abbreviations: BMI, body mass index; CON, control group; BIKE, active commuting group; MOD, moderate intensity exercise group; VIG, vigorous intensity exercise group.

### 3.2 Exercise compliance

No differences were found in the total duration of the intervention, the exercise frequency and the exercise energy expenditure across the three exercise groups. As prescribed, the exercise intensity was vigorous in VIG and moderate in MOD. The self‐selected exercise intensity was moderate in BIKE. Exercise duration per exercise day was significantly shorter in VIG compared with BIKE and MOD (Table 2).

**TABLE 2:** Exercise compliance for participants in the exercise groups

	BIKE (*n* = 14)	MOD (*n* = 28)	VIG (*n* = 18)
0–3 months			
Exercise intervention duration, days	93 (85; 107)	94 (86; 102)	93 (90; 102)
Exercise frequency, exercise days/week	4.7 (4.2; 5.1)	5.0 (4.7; 5.3)	5.1 (4.6; 5.3)
Compliance, % of prescribed days	94 (83; 102)	100 (93; 105)	103 (91; 106)^¶^
Exercise duration, min/exercise day	43 (38; 52)	51 (45; 56)	33 (32; 36)^†^, ^£^
Exercise energy expenditure, kcal/exercise day			
Women	338 (317; 354)	344 (324; 379)	323 (316; 326)
Men	487 (463; 494)	435 (429; 460)	436 (417; 469)
Total exercise energy expenditure, % prescribed kcal	103 (92; 109)	106 (102; 112)	105 (101; 107)
Exercise intensity, %VO_2_peak‐reserve	54 (46; 57)	51 (47; 53)	67 (59; 74)^‡^, ^£^
0–6 months			
Exercise intervention duration, days	188 (178; 197)	191 (182; 197)	193 (183; 198)
Exercise frequency, exercise days/week	4.4 (4.1; 4.6)	4.7 (4.2; 4.9)	4.8 (4.1; 5.2)
Compliance, % of prescribed days	88 (81; 93)	93 (84; 99)	97 (81; 103)
Exercise duration, min/exercise day	42 (38; 52)	52 (48; 59)	35 (31; 39)^†^, ^£^
Exercise energy expenditure, kcal/exercise day			
Women	341 (327; 357)	346 (328; 357)	332 (322; 346)
Men	471 (458; 510)	447 (428; 474)	441 (418; 495)
Total exercise energy expenditure, % prescribed kcal	98 (93; 101)	100 (94; 105)	101 (90; 106)
Exercise intensity, %VO_2_peak‐reserve	53 (47; 57)	50 (46; 53)	67 (59; 70)^‡^, ^£^

Data are presented as median (25th percentile; 75th percentile).

Abbreviations: %VO_2_peak‐reserve, percentage of peak oxygen uptake‐reserve; BIKE, active commuting group; MOD, moderate intensity exercise group; VIG, vigorous intensity exercise group.

† *p *< 0.05 compared to BIKE.

‡ *p *< 0.001 compared to BIKE.

$ *p *< 0.05 compared to MOD.

£ *p *< 0.001 compared to MOD.

¶ Indicate *p*‐value 0.05–0.10.

### 3.3 Body weight

Body weight was reduced in all exercise groups compared with CON at 3 and 6 months (Table 3).

**TABLE 3:** Changes in body weight, cardiorespiratory fitness and insulin sensitivity following 3 and 6 months of intervention compared with the control group

	Estimated mean differences versus CON					
	BIKE (*n* = 14)		MOD (*n* = 28)		VIG (*n* = 18)	
Body weight (kg)^a^						
3 months	−3.4 (−5.7; −1)	0.006	−2.4 (−4.5; −0.3)	0.024	−3.8 (−6.1; −1.5)	0.024
6 months	−5.1 (−7.9; −2.3)	<0.001	−3.3 (−5.7; −0.8)	0.009	−5.2 (−7.8; −2.6)	<0.001
Cardiorespiratory fitness (ml/min/kg)^a^						
3 months	3.9 (1.1; 6.8)	0.007	2.1 (−0.4; 4.5)	0.105	4.1 (1.4; 6.8)	0.003
6 months	5.1 (2; 8.2)	0.001	3.4 (0.7; 6.1)	0.014	6.3 (3.4; 9.2)	<0.001
Insulin sensitivity (%)^a^, ^b^						
3 months	9 (−12; 34)	0.424	14 (−6; 37)^c^	0.173	23 (1; 50)	0.041
6 months	29 (5; 59)	0.015	27 (6; 53)	0.010	35 (11; 64)	0.003

Abbreviations: BIKE, active commuting group; CON, control group; MOD, moderate intensity exercise group; VIG, vigorous intensity exercise group.

All results presented are estimates obtained from the linear mixed model used for the analyses. Results are presented as estimated mean differences in change and 95% confidence interval compared with CON.

^a^ Adjusted for sex.

^b^ Transformed for analysis and back‐transformed for presentation. Insulin sensitivity: glucose infusion rate measured by the hyperinsulinaemic euglycaemic clamp divided by insulin levels during the clamp.

^c^ *n* = 26.

### 3.4 Cardiorespiratory fitness

Cardiorespiratory fitness increased in all three exercise groups and was significantly higher in BIKE and VIG at both 3 and 6 months compared with CON. In MOD, the difference from CON only reached significance at 6 months (Table 3). Raw data of cardiorespiratory fitness are presented in Table S1 (supplementary material available at https://doi.org/10.6084/m9.figshare.14253104).

### 3.5 Insulin sensitivity

**FIGURE 1:**
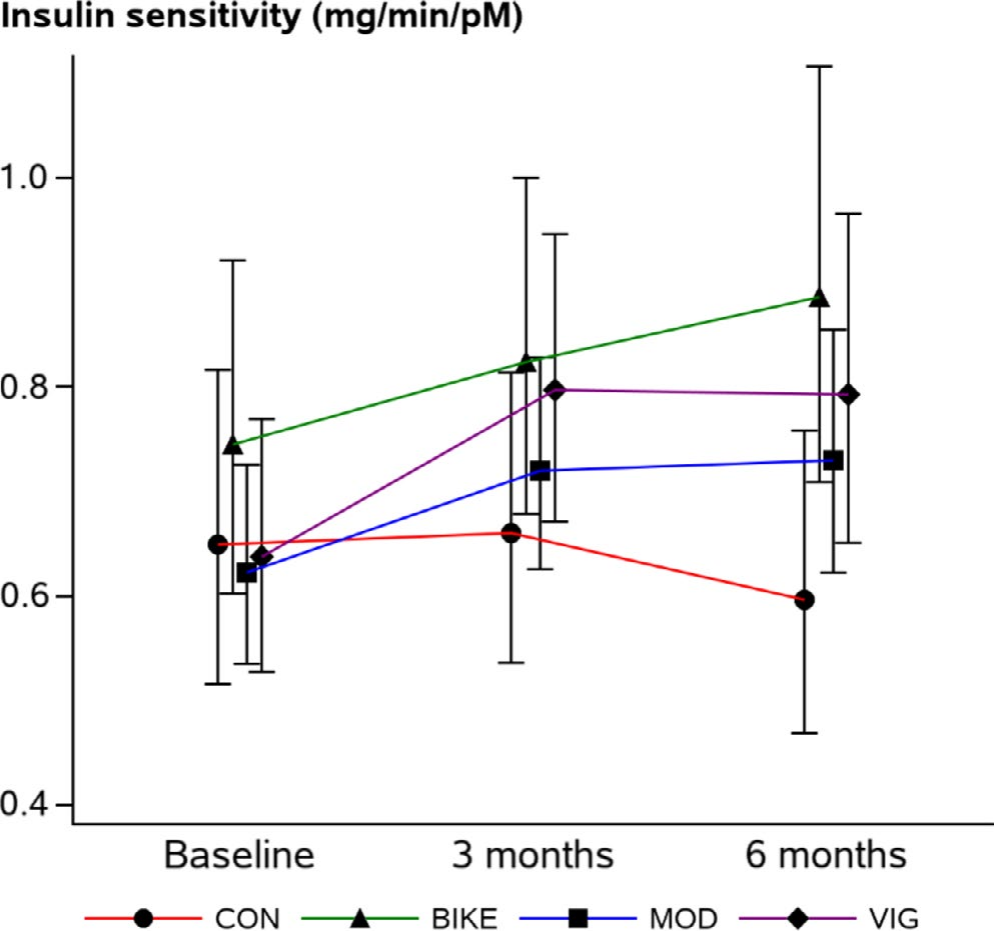
Insulin sensitivity at baseline and following 3 and 6 months of intervention. Results are presented as estimated means with 95% confidence intervals for each group at each time‐point. Adjusted for sex. Data presenting insulin sensitivity are transformed for analysis and back‐transformed for presentation. Abbreviations: CON: control group; BIKE: active commuting group; MOD: moderate intensity exercise group; VIG: vigorous intensity exercise group

Insulin sensitivity increased by 23% in VIG compared with CON at 3 months. At 6 months, insulin sensitivity was increased in all intervention groups (BIKE: 29%, MOD: 27%, VIG: 35%) compared with CON (Table 3 and Figure 1). Raw data of insulin sensitivity are presented in Table S1.

### 3.6 Protein expression

**FIGURE 2:**
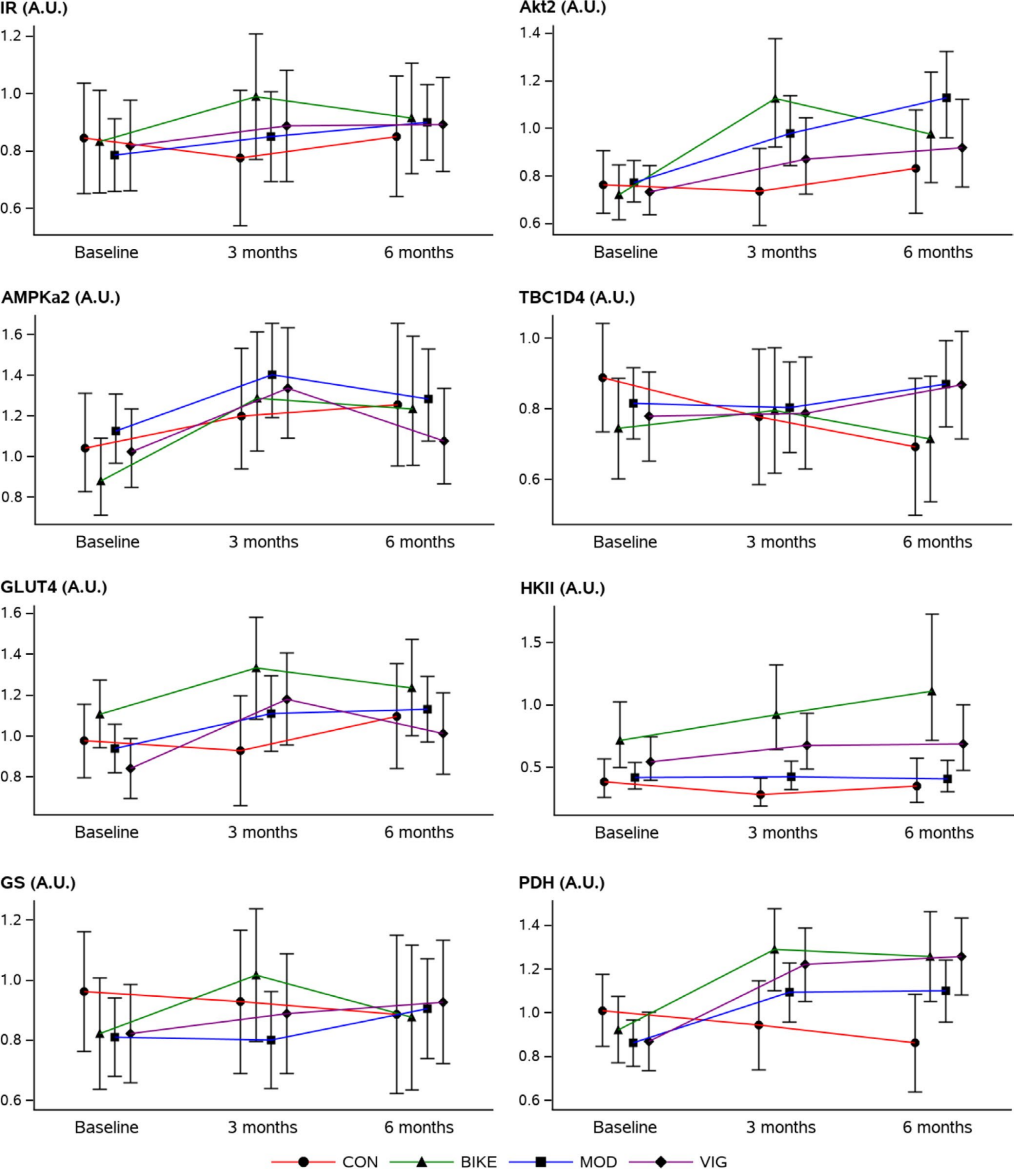
Protein expression in *m*. *vastus lateralis* at baseline and following 3 and 6 months of intervention. Results are presented as estimated means with 95% confidence intervals for each group at each time‐point. Akt2, AMPKa2 and HKII were transformed for analysis and back‐transformed for presentation. Abbreviations: Akt2: Rac‐beta serine/threonine‐protein kinase 2; AMPKα_2_: 5′‐AMP‐activated protein kinase catalytic subunit alpha‐2; A.U.: arbitrary units; BIKE: active commuting group; CON: control group; GLUT4: glucose transporter 4; GS: glycogen synthase; IR: insulin receptor; HKII: hexokinase 2; MOD: moderate intensity exercise group; PDH: pyruvate dehydrogenase; TBC1D4: TBC1 domain family member 4; VIG: vigorous intensity exercise group

Compared with the control group no statistically significant changes were observed for the expression of AMPKα_2_ and GS during the intervention. After three months of intervention we observed statistically significant increases in the expression of IR, AKT2, and HKII in the BIKE group and of GLUT4 and HKII in the VIG group; none of these changes persisted at 6 months. At 6 months only, an increased expression of TBC1D4 was found in the VIG group. The expression of PDH increased in all three exercise groups during the first 3 months of intervention and the effect was maintained from 3 to 6 months; the effect was enhanced by a decrease in CON at 6 months. No proteins were differentially expressed between VIG and MOD (Table 4 and Figure 2). Summary of the raw data for protein expression are presented in Figure S1. Representative western blots are presented in Figure S2.

**TABLE 4:** Between group comparison of changes in protein expression following 3 and 6 months of intervention

	Comparison with CON						VIG compared with MOD	
	BIKE (*n* = 14)		MOD (*n* = 28)		VIG (*n* = 18)			
IR (A.U.)^a^								
3 months	0.23 (0.02; 0.44)	0.033	0.13 (−0.05; 0.32)	0.155	0.14 (−0.06; 0.34)	0.170	0.00 (−0.16; 0.17)	0.952
6 months	0.07 (−0.22; 0.37)	0.612	0.11 (−0.15; 0.36)	0.406	0.07 (−0.21; 0.34)	0.631	−0.04 (−0.26; 0.18)	0.710
Akt2 (%)^b^								
3 months	62 (18; 122)	0.003	31 (−1; 74)	0.056	23 (−9; 66)	0.176	−6 (−27; 20)	0.596
6 months	24 (−16; 84)	0.274	34 (−5; 88)	0.092	15 (−20; 66)	0.447	−14 (−36; 15)	0.298
AMPKa2 (%)^b^								
3 months	27 (−9; 77)	0.162	8 (−20; 46)	0.603	13 (−18; 56)	0.442	5 (−20; 36)	0.729
6 months	16 (−23; 75)	0.466	−6 (−34; 35)	0.749	−13 (−41; 28)	0.481	−8 (−32; 25)	0.609
TBC1D4 (A.U.)^a^								
3 months	0.16 (−0.07; 0.40)	0.172	0.10 (−0.11; 0.31)	0.338	0.12 (−0.10; 0.34)	0.284	0.02 (−0.16; 0.20)	0.824
6 months	0.17 (−0.14; 0.47)	0.275	0.25 (−0.01; 0.52)	0.060	0.29 (0.00; 0.57)	0.048	0.03 (−0.19; 0.26)	0.769
GLUT4 (A.U.)^a^								
3 months	0.27 (−0.07; 0.62)	0.120	0.22 (−0.09; 0.52)	0.164	0.39 (0.06; 0.72)	0.023	0.17 (−0.10; 0.44)	0.221
6 months	0.01 (−0.32; 0.33)	0.962	0.07 (−0.21; 0.35)	0.621	0.05 (−0.25; 0.35)	0.747	−0.02 (−0.26; 0.22)	0.859
HKII (%)^b^								
3 months	76 (5; 195)	0.032	38 (−13; 118)	0.169	70 (4; 178)	0.036	23 (−18; 86)	0.309
6 months	69 (−14; 234)	0.125	6 (−41; 92)	0.836	39 (−26; 161)	0.308	30 (−21; 114)	0.293
GS (A.U.)^a^								
3 months	0.23 (−0.08; 0.54)	0.149	0.02 (−0.25; 0.30)	0.861	0.10 (−0.20; 0.40)	0.500	0.08 (−0.17; 0.32)	0.536
6 months	0.13 (−0.25; 0.51)	0.500	0.17 (−0.16; 0.50)	0.305	0.18 (−0.17; 0.54)	0.311	0.01 (−0.27; 0.29)	0.941
PDH (A.U.)^a^								
3 months	0.43 (0.19; 0.68)	<0.001	0.30 (0.08; 0.52)	0.008	0.42 (0.18; 0.66)	<0.001	0.12 (−0.07; 0.32)	0.220
6 months	0.48 (0.19; 0.77)	0.001	0.39 (0.13; 0.64)	0.003	0.54 (0.27; 0.81)	<0.001	0.15 (−0.06; 0.37)	0.164

All results presented are estimates obtained from the linear mixed model used for the analyses.

Abbreviations: A.U., arbitrary units; Akt2, Rac‐beta serine/threonine‐protein kinase 2; AMPK_α2_, 5´‐AMP‐activated protein kinase catalytic subunit alpha‐2; BIKE, active commuting group; CON, control group; GLUT4, glucose transporter 4; GS, glycogen synthase; HKII, hexokinase 2; IR, Insulin receptor; MOD, moderate intensity exercise group; PDH, pyruvate dehydrogenase; TBC1D4, TBC1 domain family member 4;VIG, vigorous intensity exercise group.

^a^ Results are presented as estimated mean differences in change (95% confidence interval) between the two groups being compared.

^b^ Transformed for analysis and back‐transformed for presentation; results are presented as the estimated ratio (95% confidence interval) between the relative changes from baseline to follow‐up between the two groups being compared. At 3 months, protein expression data were missing for three participants in MOD and one in VIG. At 6 months, two participants in CON, BIKE, and MOD, plus one in VIG, were missing protein expression data.

### 3.7 Associations between protein expression and insulin sensitivity

**FIGURE 3:**
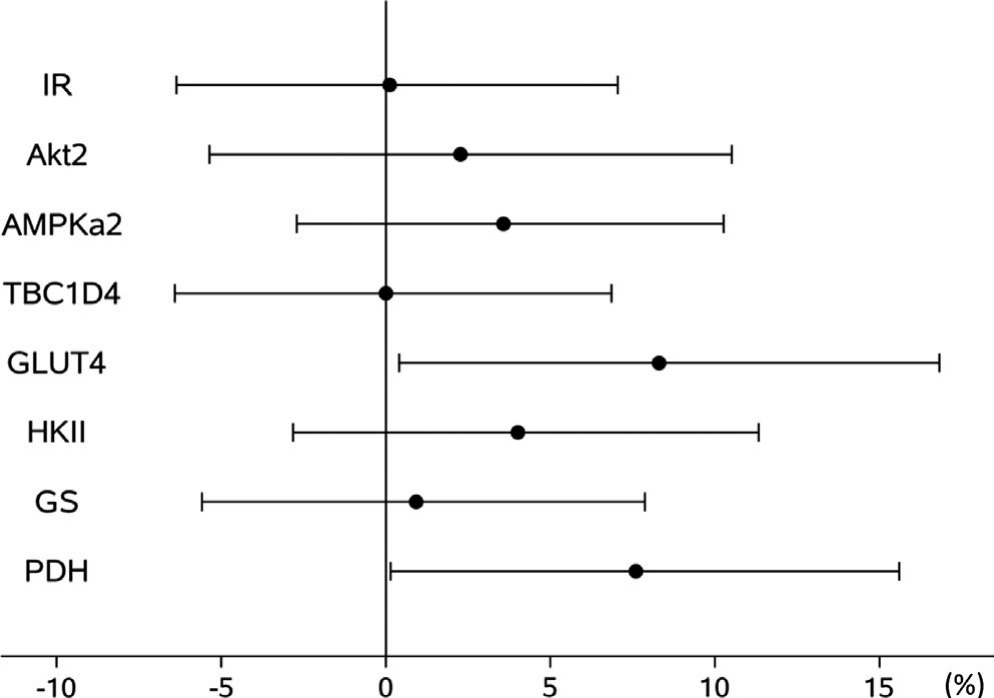
Associations between protein expression and insulin sensitivity across all time points. Results are presented as percentage change (95% confidence interval) in insulin sensitivity per 1 SD change in protein expression. Data for Akt2, AMPKα_2_ and HKII were log‐transformed for analysis. Abbreviations: Akt2: Rac‐beta serine/threonine‐protein kinase 2; AMPKα_2_: 5′‐AMP‐activated protein kinase catalytic subunit alpha‐2; GLUT4: glucose transporter 4; GS: glycogen synthase; HKII: hexokinase 2; IR: insulin receptor; PDH: pyruvate dehydrogenase; TBC1D4: TBC1 domain family member 4

Out of the eight proteins investigated the expression of GLUT4 (standardized beta 8.3%, 95% CI 0.4; 16.8, *p* = 0.039) and PDH (standardized beta 7.6%, 95% CI 0.1; 15.6, *p* = 0.046) were associated with insulin sensitivity (IR *p* = 0.972, Akt2 *p* = 0.565, AMPKα_2_ *p* = 0.266, TBC1D4 *p *= 1.000, HKII *p* = 0.253, and GS *p* = 0.785) (Figure 3 and Table S2).

## 4 DISCUSSION

Six months of exercise training induced an increase in the expression of PDH in skeletal muscle. In addition, the expression of IR, Akt2, GLUT4, and HKII was increased in one to two groups following 3 months of intervention; whereas the expression of TBC1D4 was increased in the VIG group only at 6 months. Our results did not indicate convincing differential effects of moderate and vigorous intensity exercise on key muscle proteins in the glucose metabolic pathway. Skeletal muscle protein expressions of GLUT4 and PDH were both positively associated with insulin sensitivity.

The primary aim of this study was to investigate molecular adaptations in skeletal muscle following active commuting by bike or leisure‐time exercise training in relation to insulin sensitivity. Skeletal muscle molecular adaptations to exercise training have previously been examined in smaller non‐randomized studies of 3–12 weeks duration (Biensø et al., 2015; Burgomaster et al., 2008; Christ‐Roberts et al., 2004; Dela et al., 1993; Frøsig et al., 2007; LeBlanc et al., 2004; Mortensen et al., 2013; Steenberg et al., 2019; Vind et al., 2011). This is the largest controlled study examining several selected skeletal muscle proteins related to insulin sensitivity after different types of exercise training, spanning from the muscle plasma membrane to the mitochondria. Overall, the interventions did not change the expression of IR markedly compared with control. This is consistent with previous findings in young healthy men following 3–10 weeks of single‐leg endurance exercise training (Dela et al., 1993; Frøsig et al., 2007). Thus, it appears that the expression of IR in untrained muscle is sufficient to mediate improvements in insulin sensitivity after exercise training. In our study, the expression of Akt2 was increased in BIKE following 3 months of exercise training but the effect was not persistent at 6 months. An increased expression of Akt2 has likewise been found in healthy, elderly men (Biensø et al., 2015) and in obese middle‐aged men with or without type 2 diabetes (Vind et al., 2011) following 8–10 weeks of endurance type exercise training. However, this is contradicted by the finding that 3 weeks of single‐leg endurance exercise training improved insulin sensitivity in healthy young men without changing Akt2 expression (Frøsig et al., 2007). In addition, we did not find Akt2 expression to be associated with insulin sensitivity. Thus, increased insulin sensitivity following exercise training is not always accompanied by an increased Akt2 expression.

In the distal insulin signaling cascade and the transport step, we investigated TBC1D4 and GLUT4 protein expression. In contrast to insulin sensitivity, the effects observed for TBC1D4 were primarily driven by a decrease in the control group and we found no association between TBC1D4 and insulin sensitivity. This is supported by findings in healthy young as well as elderly men following 3–12 weeks of endurance type of exercise training (Biensø et al., 2015; Frøsig et al., 2007; Steenberg et al., 2019) although one study reported a minor increase in TBC1D4 expression following 10 weeks of endurance exercise training in middle‐aged obese men with or without type 2 diabetes (Vind et al., 2011). The GLUT4 expression increased from baseline to 3 months in all exercise groups; but the change was only statistically significant in VIG. This increased GLUT4 expression was somewhat retained in all exercise groups at 6 months, but an increase in the expression in the control group meant that this did not translate into an increase compared with the controls. In coherence with the findings from a small study in humans (Dela et al., 1993), we found the GLUT4 expression to be positively associated with insulin sensitivity, underlining the importance of the size of the recruitable pool of GLUT4 for the insulin‐stimulated glucose uptake (Maarbjerg et al., 2011). Previous studies have shown an increase in GLUT4 expression after 3–10 weeks of endurance exercise training in both healthy young men, with and without overweight (Biensø et al., 2015; Dela et al., 1993; Frøsig et al., 2007; Nordby et al., 2012) and in middle‐aged men with insulin resistance or type 2 diabetes (Christ‐Roberts et al., 2004) supporting our findings at 3 months. Nevertheless, unchanged expression of GLUT4 has also been found following endurance exercise training for 12 weeks (Steenberg et al., 2019). Using muscle‐specific AMPKα_1_α_2_ knockout mice, we have previously provided evidence to support that the AMPKα_2_‐associated trimer protein complex phosphorylates TBC1D4 to enhance insulin‐stimulated glucose uptake in skeletal muscle (Kjøbsted et al., 2015, 2017, 2019). However, in the present study we did not find any changes in AMPKα_2_ expression in any of the training groups compared with controls. This is in agreement with studies in lean, younger men following 12 weeks of endurance training (Mortensen et al., 2013; Steenberg et al., 2019) and suggests that exercise training‐induced improvements in peripheral insulin sensitivity is independent of enhanced AMPKα_2_ expression. It has been reported that AMPKα_2_β_2_γ_1_‐associated activity may increase following exercise training even though AMPKα_2_ protein expression does not (Mortensen et al., 2013). However, based on our findings in rodents such increase in AMPKα_2_β_2_γ_1_‐associated activity likely does not contribute to the regulation of muscle insulin sensitivity (Kjøbsted et al., 2015, 2017).

The intracellular metabolism of glucose in skeletal muscle is affected by insulin as indicated by increased glucose oxidation and storage of glucose in the form of glycogen during insulin stimulation (Kelley et al., 1990). GS and PDH are considered the rate‐limiting enzymes for glycogen synthesis and glucose oxidation, respectively (Holness & Sugden, 2003; Skurat et al., 1996). However, before glucose is partitioned towards oxidation or storage, it needs to be phosphorylated by HKII, subsequently trapping it as glucose‐6‐phosphate allowing more glucose to enter the cell (Wasserman et al., 2011). Together this suggests an important regulatory role of GS, PDH, and HKII for insulin‐stimulated glucose uptake. We did not observe any changes in GS protein expression during and after the exercise intervention. This contrasts with most (Mortensen et al., 2013; Steenberg et al., 2019; Vind et al., 2011) but not all (Frøsig et al., 2007) studies showing increased GS expression in skeletal muscle following a period of endurance exercise training in young, lean men as well as in middle‐aged, obese men with or without type 2 diabetes. This discrepancy cannot be attributed to differences in training intensity as this is fairly comparable across the different studies. Based on our study, elevated GS expression does not seem to be a prerequisite for improved peripheral insulin sensitivity following exercise training. This is consistent with findings in transgenic mice showing no further increase in insulin‐stimulated glucose uptake in skeletal muscle overexpressing active GS (Fogt et al., 2004). HKII is regarded as one of the most responsive proteins to exercise training and is found to increase in most studies (Biensø et al., 2015; Frøsig et al., 2007; Steenberg et al., 2019; Vind et al., 2011). Furthermore, overexpression of HKII protein in mouse skeletal muscle has been shown to increase peripheral insulin sensitivity (Fueger et al., 2004), which suggests a role of HKII for training‐induced improved peripheral insulin sensitivity. However, we did not find a convincing increase in the expression of HKII compared to previous studies, nor did we find a strong association with insulin sensitivity. Thus, the improvements in insulin sensitivity after exercise training observed in our exercise groups are at least only partly explained by changes in HKII expression.

We found an increased expression of PDH in all exercise groups following the intervention. Only a few other studies have investigated PDH expression in human skeletal muscle following exercise training. In agreement with our study, these show a minor increase following 8 weeks of endurance type of exercise training in healthy young and elderly men (Biensø et al., 2015; LeBlanc et al., 2004) and following 6 weeks of endurance or sprint interval training in healthy young women and men (Burgomaster et al., 2008). In addition, we found that the expression of PDH was significantly associated with insulin sensitivity. This could suggest a potential role of PDH in the regulation of peripheral insulin sensitivity and that exercise training‐induced improvements in peripheral insulin sensitivity may in part be mediated by an increased expression of PDH in skeletal muscle. Since PDH is considered the rate limiting enzyme for glucose oxidation (Holness & Sugden, 2003), it can be hypothesized that an increased expression of PDH will enhance glycolytic flux, which may drive glucose uptake into skeletal muscle during insulin stimulation. The role of PDH in regulating insulin‐stimulated glucose uptake in skeletal muscle has been investigated in a few animal studies. In mice, knockout of PDH in skeletal muscle does not affect insulin‐stimulated glucose uptake in isolated muscle nor does it compromise whole body glucose tolerance (Svensson et al., 2018). Moreover, mice lacking pyruvate dehydrogenase kinase 2 and 4 (PDK2 and PDK4), which results in constitutively active PDH, display reduced insulin‐stimulated glucose uptake in skeletal muscle compared with wild type mice. However, this could be explained by elevated levels of muscle lipids in PDK‐deficient mice leading to muscle insulin resistance (Rahimi et al., 2014). To circumvent confounding factors often observed in transgenic animals, Small and co‐workers recently investigated the effect of acute PDH activation by dichloroacetate (DCA; PDK inhibitor) infusion to enhance insulin‐stimulated muscle glucose uptake in rats (Small et al., 2018). Although acute pharmacological activation of PDH by DCA infusion increased glucose oxidation it did not seem to enhance insulin‐stimulated glucose uptake in skeletal muscle. Collectively, this suggests that PDH is not involved in regulating muscle glucose uptake during insulin stimulation in rodents, but rather acts to balance the oxidative/non‐oxidative metabolism of glucose. Since these studies in rodents do not support our findings suggesting a role of PDH for regulating muscle insulin sensitivity in humans, future studies investigating PDH in human skeletal muscle are needed. Meanwhile, a recent study in individuals with obesity, NAFLD and T2D reported that exercise‐induced changes in peripheral insulin sensitivity were associated with changes in glucose oxidation rather than non‐oxidative glucose disposal rates (Mancilla et al., 2021). Together these findings support that PDH regulates insulin sensitivity in human skeletal muscle and therefore may function as a therapeutic target for the treatment of skeletal muscle insulin resistance. However, reverse causality, suggesting that improved insulin sensitivity facilitates an enhanced glucose oxidation, cannot be ruled out and further research is warranted.

We found that changes in protein expression following moderate and vigorous exercise were comparable. If differences in expression do exist for the selected eight proteins it will likely require a larger number of participants to detect. Exercise training is also associated with increased mitochondrial content in skeletal muscle. However, it appears that training volume rather than exercise intensity is the most important determinant of exercise training‐induced increases in mitochondrial content (Granata et al., 2018). This may also apply to proteins in the glucose metabolic pathways, which could explain the lack of difference in muscle protein expression following moderate and vigorous intensity exercise training of similar volume.

The current study is one of the largest studies investigating molecular adaptations in skeletal muscle and insulin sensitivity following exercise training. Furthermore, it has a longer duration, contains repeated measurements and includes a non‐exercising control group. Especially the latter has been a limitation in previous studies, and it enables us to isolate the exercise training effects from the effect of time and study participation. A limitation of our study is the lack of muscle biopsies obtained in the insulin‐stimulated state; this only allowed us to investigate changes in protein expression by exercise training and not the regulation of these during insulin stimulation. Although PDH activity does not seem to differ in skeletal muscle from trained and untrained subjects (Gudiksen et al., 2017), measurements of PDH activity in trained skeletal muscle during insulin stimulation could further elucidate the potential role of PDH for regulating exercise‐induced improvements in peripheral insulin sensitivity.

Additional important methodological limitations to consider are the exploratory nature of the study and the number of tests performed, which prompted us to use a fixed level for statistical significance based on a *p*‐value. It should be noted, however, that when the posterior probability of a true effect is large, for instance based on prior research, the *p*‐value is of limited value and might lead to rejection of actual biological effects (Nuzzo, 2014). Conversely, when the null hypothesis is true, the chance of obtaining a low *p*‐value is as likely as obtaining a high *p*‐value, and therefore focusing too much on a low *p*‐value may lead to flawed conclusions (Nuzzo, 2014). Given the number of tests performed, the expected number of false positive tests is 3 for the analysis of intervention effects on protein expression and 0.4 for the association analysis. As always, our findings, especially those related to PDH, need to be reproduced in trials designed for this purpose.

In conclusion, 6 months of leisure‐time exercise training or active commuting by bike were accompanied by an increased expression of PDH in skeletal muscle when compared with control. No differential effects were observed in skeletal muscle protein expression following moderate and vigorous intensity exercise training. The expression of both GLUT4 and PDH was positively associated with insulin sensitivity. The positive association and the increase in the expression of PDH after exercise training points towards a role for PDH in the training‐induced enhancement of peripheral insulin sensitivity.

## 5 Disclaimers

The funders had no role in study design, data collection and analysis, decision to publish, or preparation of the manuscript.

## Conflict of interest

The authors declare they do not have anything to disclose regarding conflict of interest with respect to this manuscript.

## Author contributions

M.B.B., J.S.Q., A.S.G., M.R. and B.S. conceived and designed the GO‐ACTIWE study and performed the practical experiments. L.B. performed the laboratory work related to the muscle biopsies with supervision from R.K. and J.W. L.B. and M.B.B. analysed data and L.B., M.B.B., R.K., K.F., and B.S. interpreted results and experiments. L.B. wrote the first draft of the paper and all other authors edited and revised the manuscript and approved the final version.

## Supporting information



Supplementary Material
