# Supplementary material for: Effect of exercise training on skeletal muscle protein expression in relation to insulin sensitivity: Per‐protocol analysis of a randomized controlled trial (GO‐ACTIWE)
Source: Physiol Rep. 2021 May 27;9(10):e14850. doi: 10.14814/phy2.14850 (PMC8157763; doi:10.14814/phy2.14850)
Supplement: Supplementary file 1 — Supplementary Material [file PHY2-9-e14850-s001.pdf]

Supplementary:

Table S1: Summary of raw data for body weight, cardiorespiratory fitness and insulin sensitivity at baseline and following 3 and 6 months of intervention

	CON (n=12)	BIKE (n=14)	MOD (n=28)	VIG (n=18)
**Body weight (kg)**				
Baseline	93.0 (90.3; 101.5)	90.0 (81.5; 100.4)	92.9 (79.7; 97.2)	90.7 (81.7; 104.7)
3 months	96.4 (89.6; 105.8)	87.2 (81.3; 97.9)	92.1 (78.5; 98.2)	92.1 (79.5; 102.1)
6 months	97.9 (88.1; 106.9)	86.0 (81.8; 95.2)	92.1 (77.9; 98.1)	92.2 (77.8; 101.4)
**Cardiorespiratory fitness (mL/min/kg)**				
Baseline	30.5 (23.8; 35.6)	30.8 (27.3; 34.1)	30.8 (27.0; 33.4)	30.6 (26.2; 32.4)
3 months	29.5 (25.5; 31.2)	34.4 (29.8; 40.0)	33.1 (28.3; 36.1)	34.0 (31.6; 36.7)
6 months	29.0 (23.7; 33.2)	35.8 (30.6; 40.9)	34.4 (28.7; 37.5)	35.6 (31.6; 38.9)
**Insulin sensitivity (mg/min/pM)**				
Baseline	0.67 (0.49; 0.82)	0.66 (0.47; 0.98)	0.66 (0.52; 0.75)*	0.63 (0.47; 0.96)
3 months	0.71 (0.46; 0.85)	0.74 (0.59; 1.14)	0.75 (0.60; 0.98)**	0.84 (0.55; 1.21)
6 months	0.69 (0.40; 0.85)	0.89 (0.66; 1.02)	0.85 (0.50; 1.10)	0.79 (0.58; 1.14)

Data are presented as median (25th and 75th percentile). Insulin sensitivity: glucose infusion rate measured by the hyperinsulinaemic euglycaemic clamp divided by insulin levels during the clamp. * n= 27. ** n=26. Abbreviations: CON: control group; BIKE: active commuting group; MOD: moderate intensity exercise group; VIG: vigorous intensity exercise group

Table S2: Association analysis between insulin sensitivity and protein expression across all time points

**IR**	0.1 (-6.4; 7.0)	0.972
**Akt2**	2.3 (-5.4; 10.5)	0.565
**AMPKa2**	3.6 (-2.7; 10.3)	0.266
**TBC1D4**	0.0 (-6.4; 6.9)	1.000
**GLUT4**	8.3 (0.4; 16.8)	0.039
**HKII**	4.0 (-2.8; 11.3)	0.253
**GS**	0.9 (-5.6; 7.9)	0.785
**PDH**	7.6 (0.1; 15.6)	0.046

Results are presented as percentage change (95% confidence interval) in insulin sensitivity per 1 SD change in protein expression. Data for Akt2, AMPKa2 and HKII were log-transformed for analysis. IR: Insulin receptor; Akt2: Rac-beta serine/threonine-protein kinase 2; AMPKα2: 5´-AMP-activated protein kinase catalytic subunit alpha-2; TBC1D4: TBC1 domain family member 4; GLUT4: Glucose transporter 4; HKII: hexokinase 2; GS: glycogen synthase; PDH: pyruvate dehydrogenase.

Figure S1: Protein expression in the groups at baseline (blue) and following 3 (red) and 6 (green) months of intervention (raw data)

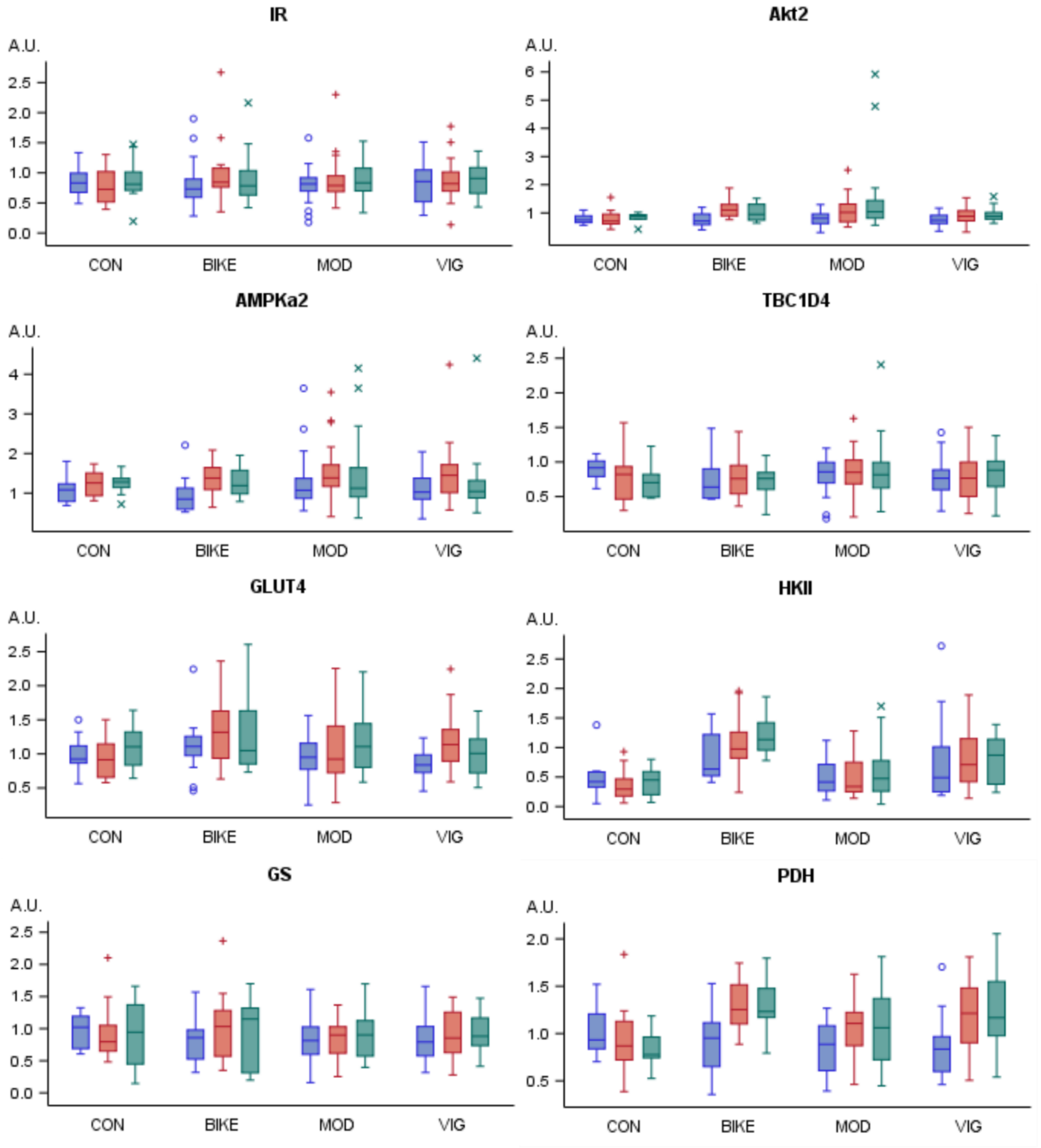

The box indicates the interquartile range, the line the median and the whiskers the 5th and 95th percentile in arbitrary units. Individual markers indicate outliers (1.5 interquartile ranges above the 75th percentile). At 3 months, protein expression data were missing for three participants in MOD and one in VIG. At 6 months, two participants in CON, BIKE and MOD, plus one in VIG, were missing protein expression data. Abbreviations: Akt2: Rac-beta serine/threonine-protein kinase 2; AMPKα2: 5´-AMP-activated protein kinase catalytic subunit alpha-2; BIKE: active commuting group; CON: control group; GLUT4: Glucose transporter 4; GS: glycogen synthase; IR: Insulin receptor; HKII: hexokinase 2; MOD: moderate intensity exercise group; PDH: pyruvate dehydrogenase; TBC1D4: TBC1 domain family member 4; VIG: vigorous intensity exercise group.

Figure S2: Representative Western blot images

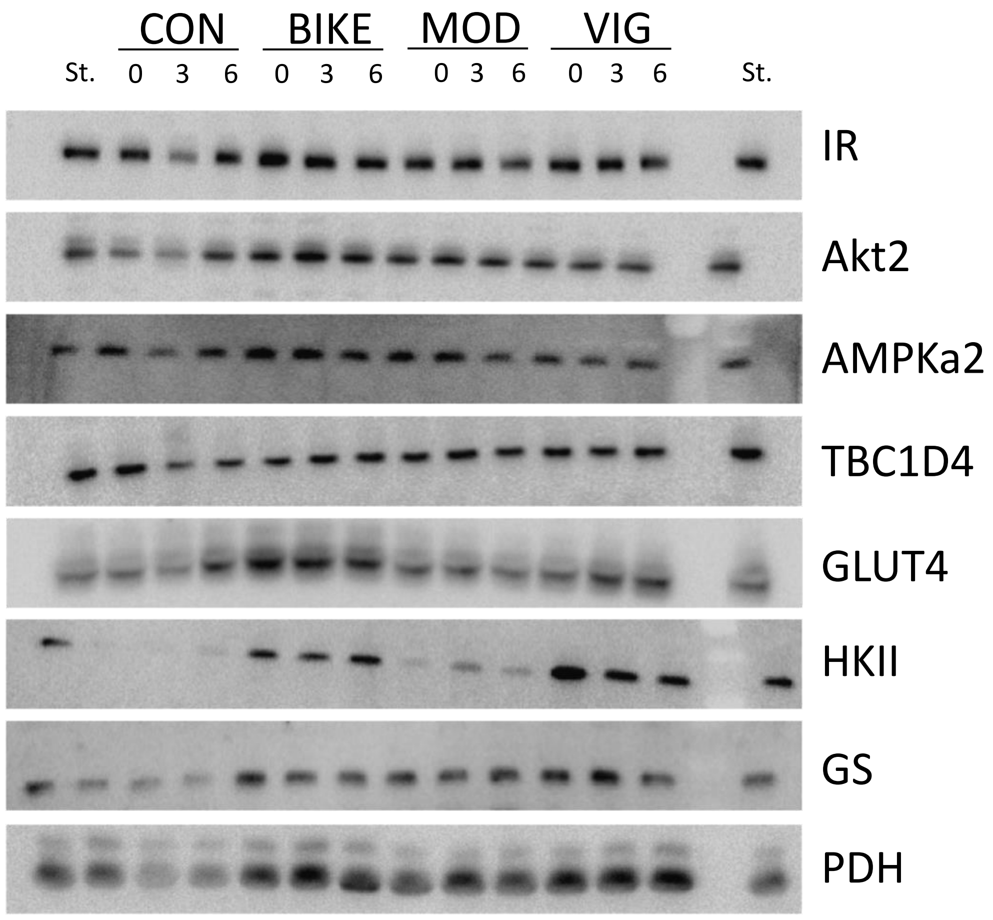

Protein expression of IR, Akt2, AMPKa2, TBC1D4, Glut4, HKII, GS and PDH in m. vastus lateralis.
Abbreviations: Akt2: Rac-beta serine/threonine-protein kinase 2; AMPKa2: 5´-AMP-activated protein kinase catalytic subunit alpha-2; BIKE: active commuting group; CON: control group; GLUT4: Glucose transporter 4; GS: glycogen synthase; IR: Insulin receptor; HKII: hexokinase 2; MOD: moderate intensity exercise group; PDH: pyruvate dehydrogenase; St.: standard sample; TBC1D4: TBC1 domain family member 4; VIG: vigorous intensity exercise group.
